# Early Second-Trimester Serum MicroRNAs as Potential Biomarker for Nondiabetic Macrosomia

**DOI:** 10.1155/2014/394125

**Published:** 2014-10-27

**Authors:** Lingmin Hu, Jing Han, Fangxiu Zheng, Hongxia Ma, Jiaping Chen, Yue Jiang, Hua Jiang

**Affiliations:** ^1^Changzhou Maternity and Child Health Care Hospital, Nanjing Medical University, Changzhou, Jiangsu 213003, China; ^2^Department of Epidemiology and Biostatistics, State Key Lab of Reproductive Medicine, School of Public Health, Nanjing Medical University, Nanjing 210029, China

## Abstract

*Background*. Macrosomia has become a worldwide problem with the rapid economic growth in the past few years. However, the detailed mechanism of how the macrosomia happened remains unknown. Growing evidence indicates that miRNAs are involved in maintaining metabolic homeostasis. We hypothesized that serum miRNAs are potential biomarkers for macrosomia.* Methods*. We performed miRNAs profiling using TLDA chips in the discovery phase in two pooled samples from 30 cases and 30 controls, respectively. Individual qRT-PCR was conducted for the discovery phase samples. To confirm the results, we detected the miRNAs which were differentially expressed in the microarray assays and individual qRT-PCR in external validation phase with another 30 cases and 30 controls.* Results*. In the discovery stage, miR-194 and miR-376a expression levels were significantly different between macrosomia group and controls (*P* = 0.048 for miR-194 and *P* = 0.018 for miR-376a, resp.). Further evaluation of the two miRNAs on a total of 120 serum samples showed that the miR-376a remains significantly lower in macrosomia (*P* = 0.032). Receiver operating characteristic curve analyses showed that the area under curve for miR-376a was 67.8% (sensitivity = 96.7% and specificity = 40.0%).* Conclusions*. Serum miR-376a may serve as a potential noninvasive biomarker in detecting macrosomia.

## 1. Introduction

Fetal macrosomia now is usually defined as having a fetal weight of above the 90th percentile, a birth weight (BW) of above 4000 g or 4500 g, or a birth weight of over +2 standard deviation of the mean birth weight by gestational age [1, 2]. With rapid economic growth in China, the rate of macrosomia drastically rose from 6.00% in 1994 to 7.83% in 2005 [3]. Macrosomia is not only associated with numerous perinatal and maternal complications such as shoulder dystocia, brachial plexus injury, prolonged labor, but also associated with long-term effects on type 2 diabetes mellitus, hypertension, and obesity in adulthood [4–6]. The pathophysiology of macrosomia is related to the associated maternal or fetal condition that accounts for its development. In general, maternal overweight and the related metabolic changes, such as diabetes mellitus type 2 and GDM, seem to be crucially important for macrosomia [7, 8]. There have been proposed interventions based on the evidence for the management of diabetic macrosomia. Nondiabetic macrosomia is still an obstetric dilemma as there is no clear consensus regarding its antepartum prediction and management, with the mechanism of nondiabetic macrosomia still remaining poorly known.

MicroRNAs (miRNAs) are a class of small noncoding RNAs that can repress translation or promote degradation of target mRNAs of complementary sequences. Deregulation of miRNAs is known to be involved in multiple processes including cell proliferation, apoptosis, and cell-cycle regulation, inflammation, and invasion in various diseases. MicroRNAs have been found in tissues, serum/plasma, and other body fluids, in a stable form that is protected from endogenous RNase activity. These unique characteristics of miRNAs may provide a useful biomarker for supplemental diagnosis. Several studies have highlighted the significance of miRNAs in maintaining metabolic homeostasis, and thus regulation of these miRNAs could serve as potential therapeutics in metabolic disorders [9, 10]. miRNAs are involved in the pathogenesis of diabetes mellitus and that a number of miRNAs have been reported to be differently expressed in pancreatic *β*-cells and/or adipose tissue of animal models of type 1 or type 2 diabetes [11]. Specifically, miRNAs are required for pancreatic development and the regulation of glucose stimulated insulin secretion [12–14]. In the previous work, we had demonstrated that miRNAs in placentas had the potential to be new biomarkers in patients with macrosomia [15]. However, no study has investigated circulating miRNA expression as a potential biomarker for macrosomia.

In this study, we hypothesized that circulating serum miRNAs could serve as candidate biomarkers for predicting macrosomia in relatively early pregnancy. To address this hypothesis, we systematically screened serum miRNAs by using the TaqMan low density array (TLDA) chips followed by individual quantitative reverse transcriptase polymerase chain reaction (qRT-PCR) assays. We also performed external validations by using individual qRT-PCR assays to find a class of serum miRNAs as biomarkers for macrosomia prediction.

## 2. Materials and Methods

### 2.1. Study Design and Study Population

All of the subjects were recruited at the Changzhou Maternity and Child Health Care Hospital affiliated to Nanjing Medical University from April 1, 2011, to Jun 30, 2012. The macrosomia neonates were defined as those neonates whose BW were equal to or greater than 4000 g in this study. Those neonates with BW between 2500 and 4000 g were classified as controls. We designed a retrospective nest case-control study to determine whether serum miRNAs profiling could predict macrosomia. All pregnant women provided blood samples when they received prenatal care at 16–20 gestational weeks. The mothers had no gestational diabetes mellitus during pregnancy, confirmed by negative results of oral glucose tolerance test of 75 g in 24–28 gestational weeks. To control sample heterogeneity, exclusion criteria also included diabetes history, preterm labor, preeclampsia, multiple gestations, other complications during pregnancy, and those found to have BMI > 26, age ≥ 35 years, and infant BW < 2500 g. Cases and controls were frequency matched for pregnant women's age, body mass index (BMI), and pregnant weeks at the time of blood collecting and infant gender.

Overall, a total of 120 participants with two phases were included in this study. To detect the general signatures of miRNAs for the prediction of macrosomia, we performed miRNA profiling using TLDA chips in the discovery phase in two pooled samples from 30 cases and 30 controls, respectively. Individual qRT-PCR was conducted for the discovery phase samples to further filter signals of the screened miRNAs due to heterogeneity in the subjects. To confirm the results, we detected the miRNAs which were differentially expressed in the microarray assays and individual qRT-PCR in external validation phase with another 30 cases and 30 controls. This study was approved by the Human Research Ethics Committees of Changzhou Maternity and Child Health Care Hospital affiliated to Nanjing Medical University, and a written informed consent was also obtained from each participant. Clinical information and birth outcomes were collected from the obstetric electronic medical records.

### 2.2. Serum Preparation and RNA Extraction

All of the participants were genetically unrelated, ethnic Han Chinese, and donated 5 mL of venous blood when they received prenatal care at 16–20 weeks of gestation. The whole blood was separated into serum and cellular fractions by centrifugation at 2,770 g for 10 min, followed by 20,660 g for 15 min to completely remove cell debris. The supernatant serum was stored at −80°C until analysis.

Total RNA containing small RNA was extracted from serum samples by using miRVana PARIS Kit (Applied Biosystems Inc, CA, USA) according to the manufacturer's protocol. C. elegans miRNAs were lacking of sequence homology to human miRNAs and missing empiric hybridization to human miRNA probes on miRNA microarrays. To control variations in RNA extraction and/or purification procedures, we routinely spiked in synthetic C. elegans miR-39 (cel-mir-39) (Takara, Japan) to a final concentration of 10^−4 ^pmol/uL to each denatured sample [16, 17].

### 2.3. TLDA Chip Assays and qRT-PCR

In the discovery phase, each serum sample was pooled equally before RNA isolation with a total amount of 600 *μ*l that was used for the TLDA assay (TaqMan Human miRNA Array Set v3.0; Applied Biosystems Inc., CA, USA). After total RNA isolation, Megaplex RT reactions and preamplification reactions were performed according to the manufacturer's protocol. Then, 75 *μ*l 0.1 × TE was added to preamplification product, and 9 mL diluted preamplification product was used to perform the RT-PCRs by dispensing 100 *μ*l of the PCR mix into each port of the TLDA chip. The default PCR procedure was used and the analysis was performed by using RQ manager software (Applied Biosystems Inc.). Peltier and Latham established a method to find normalizer of microRNA expression levels [18]. According to our lab experience, by testing large sample, we selected three genes (MammU6, hsa-mir-16, and hsa-mir-24) as putative reference normalizer using Peltier and Latham's way [18]. Many studies have selected miR-24 as a normalizer in miRNA expression experiment [19–21]. In this research, miR-24 also was selected considering its lowest variance of CT value in these three genes. ΔCT and ΔΔCT were calculated using the following mathematical formula: ΔCT = CT_sample_ − CT_miR-24_, ΔΔCT = ΔCT_case_ − ΔCT_control_. Differentially expressed miRNAs were chosen as candidates for further confirmation by individual qRT-PCR according to the following two items: (i) having a CT value of <35 in at least one pool to ensure the enough abundance of miRNA for detection and (ii) |ΔΔCT| > 2, which means four-fold altered expression between the cases and healthy controls to ensure favorable discriminative ability.

For qRT-PCR, equal volume of serum sample was processed in each step from serum purification to qRT-PCR. The total RNA was reversely transcribed to complementary DNA using TaqMan miRNA RT Kit and stem-loop RT primers (Applied Biosystems Inc.). The quantitative detection of miRNA was performed using the TaqMan PCR kit as implemented on the ABI 7900 Real-Time PCR System (Applied Biosystems Inc.). The reactions were initiated in a 384-well optical plate at 95°C for 5 min, followed by 40 cycles of 95°C for 15 s and 60°C for 1 min. Each plate contains equal number of cases and controls, which run the RT-PCR for target miRNAs and cel-miR-39 simultaneously. All reactions were run in triplicate. The CT values were determined using the fixed threshold settings. The relative expression levels of target miRNAs were determined by the equation 2^−ΔCT^, in which ΔCT = CT_sample_ − CT_cel-39_.

### 2.4. Statistical Analysis

Demographic and clinical characteristics among groups were compared by *χ* ^2^ test or Student-*t* test. Univariate and multiple logistic regression models were used for analysis. Receiver operating characteristic (ROC) curves were constructed in the controls of discovery phase to obtain the optimal cutoff values for candidate miRNA. The area under curve (AUC) value and 95% confidence intervals (CI) were calculated to determine the specificity and sensitivity. We set the optimal cutoff value as the threshold to code the expression level of the corresponding miRNA to each sample as 0 and 1. The risk score of each miRNA was calculated using the weights by the regression coefficient that was derived from the univariate logistic regression analysis of candidate miRNA. All the statistical analyses were performed with Stata version 9.2 (Stata Corporation, College Station, TX, USA). A *P* value < 0.05 was considered statistically significant, and all tests were two tailed.

## 3. Results

The characteristics of participants are summarized in Table 1. There were no significant differences in maternal age, gestational week, and maternal weight at delivery and gestational week, maternal weight, and maternal BMI when blood was collected between the two groups. The BW of neonates with macrosomia was 4202.25 ± 193.08 g while that in the control group was 3379.00 ± 280.26 g.

In this study, we systematically screened the miRNA profile in serum from the mothers of macrosomia cases and healthy controls using 762 probes that represented both characterized and novel miRNAs. We found 16 miRNAs differentially expressed (2 upregulated and 14 downregulated) among macrosomia cases compared with controls (ΔΔCT > 2-fold) (Table 2). Based on both scientific and applicable considerations, we selected miRNAs that had at most 35 of CT value in at least one pool by TLDA for further individual qRT-PCR confirmation. As a result, 8 miRNAs were identified and subjected to individual qRT-PCR analysis on 60 discovery-stage samples. In the discovery stage, two miRNAs (miR-194 and miR-376a) expression levels were significantly different between cases and controls (*P* = 0.048 for miR-194 and *P* = 0.018 for miR-376a, resp.) (Table 3). To further evaluate the diagnostic value of miR-194 and miR-376a, we measured the two miRNAs expression levels on a total 120 serum samples, including the external phase samples. The levels of the miR-376a remain significantly lower in serum samples of macrosomia (*P* = 0.032), while the expression level of the miR-194 showed no difference between macrosomia cases and controls (*P* = 0.497) (Table 4).

**Figure 1:**
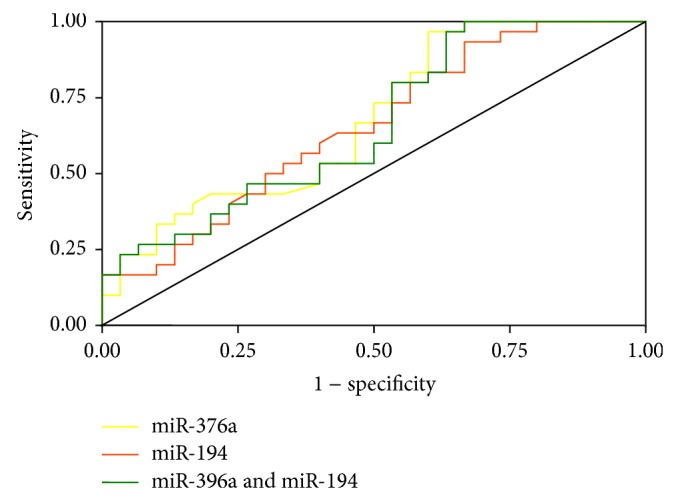
Receiver operating characteristic curve of the miR-376a and miR-194 signatures to predict nondiabetic fetal macrosomia; yellow line: miR-376a (AUC = 67.8%, sensitivity = 96.7%, and specificity = 40.0%); red line: miR-194 (AUC = 65.4%, sensitivity = 93.3%, and specificity = 33.3%); green line: miR-376a and miR-194 (AUC = 65.9%, sensitivity =96.7%, and specificity = 36.7%).

We further plotted the ROC curves for macrosomia predicting. The AUC analysis was only performed in the samples from the discovery and internal validation stages (30 pairs). ROC curve analyses showed that the AUC for miR-376a was 67.8% (sensitivity = 96.7% and specificity = 40.0%) (Figure 1).

## 4. Discussion

Circulating miRNAs have been extensively investigated as novel and noninvasive diagnostic and prognostic markers. Most published studies have been focused on different types of cancer [22–24]. In this study, we systematically screened the miRNA profile in early second-trimester serum from the mothers of macrosomia cases and healthy controls. At last, we found that signatures of the aberrant miR-376a were expressed in early second-trimester serum samples of macrosomia pregnant women.

To date, few studies have researched into the relationship between miR-376a and miR-194 and metabolic disease. However, many studies revealed that miR-376a or miR-194 has influence on tumors, such as breast cancer, pancreatic ductal adenocarcinoma, and hepatocellular carcinoma [25–27]. Apoptosis is a fundamental mechanism for maintaining homeostasis by removing dangerous and unnecessary cells. However, adipocytes are resistant to apoptosis because of high levels of Akt/protein kinase B and the antiapoptotic factor Bcl-2 [28]. Pescador et al. employed miRNA PCR panels to screen serum levels of miRNAs in pooled samples from four groups (type 2 diabetic patients, obese patients, obese patients with type 2 diabetes, and healthy controls). They found miR-376a has potential as a predictive biomarker in obesity. Use of miR-376a provides a powerful predictive tool for distinguishing obese patients from normal healthy controls, diabetic patients, and obese diabetic patients [29]. Adipocytes could be removed through apoptotic mechanisms in some pathological conditions such as obesity. The induction of apoptosis in adipocytes, by regulating miR-376a, could be a possible method to reduce the adipocyte number [29]. This result supported our study that miR-376a was a significantly predictive tool for macrosomia. Maternal diet during pregnancy and gestation has long-term effects on the offspring. As to miR-194, Zhang et al. found that maternal high fat fed offspring had markedly reduced expression of miR-194 when compared with that fed with standard chow diet [30]. miR-194 also was found remarkably decreased in diabetic mice [31]. However, the results are inconsistent and inconclusive. Guo et al. found that miR-194 was significantly downregulated in intrauterine growth restriction neonate [32]. In our study, we only detected that the miR-194 was remarkably decreased in macrosomia group in discovery phase, while no consistent result was found in all subjects. Further studies are recommended to verify these results.

Our study had a number of strengths. First of all, we collected blood samples of pregnant women at their 16–20 gestational weeks. Neonate body weight increased drastically mainly at the late pregnant stage. Increased mother's body weight in late pregnant will result in serious possibility of macrosomia. It shed light on the possible effects of earlier intervention and more aggressive treatment on maternal and fetal outcomes. Moreover, all participants are with no gestational diabetes mellitus during pregnancy or diabetes history which may reduce potential selection bias and control the confounding factor of abnormal blood glucose level. However, the sample size of the external validation was relatively small, which may present underpowered results.

## 5. Conclusion 

We demonstrated that the serum miR-376a was differentially expressed between macrosomia cases and controls, while the clinical application of these miRNAs in predicting macrosomia still needs further investigation and optimization.

## Figures and Tables

**Table 1:** Characteristics of the study population.

Variable	Discovery phase				All participants			
	Case (*n* = 30)	Control (*n* = 30)	*T*	*P*	Case (*n* = 60)	Control (*n* = 60)	*T*	*P*
Birth weight (g)	4223.00 ± 187.99	3419.33 ± 277.64	−13.13	<0.001	4202.25 ± 193.08	3379.00 ± 280.26	18.74	<0.001
Maternal age (y)	26.47 ± 2.49	26.93 ± 2.57	0.71	0.478	26.73 ± 2.62	26.45 ± 2.42	0.62	0.539
Gestational week (w) when blood was collected	17.60 ± 0.92	17.55 ± 0.91	−0.20	0.842	17.41 ± 0.83	17.493 ± 0.83	−0.56	0.580
Gestational week (w) of delivery	39.40 ± 0.82	39.19 ± 0.71	−1.06	0.293	39.35 ± 0.90	39.128 ± 0.79	1.43	0.154
Maternal weight (kg) when blood was collected	58.82 ± 6.81	57.93 ± 7.05	0.49	0.623	58.56 ± 5.61	57.642 ± 6.15	0.85	0.396
Maternal BMI (kg/m^2^) when blood was collected	22.11 ± 2.15	22.30 ± 2.54	0.31	0.761	22.17 ± 1.77	21.941 ± 2.12	0.64	0.527
Maternal weight (kg) of delivery	75.28 ± 7.79	72.37 ± 7.70	1.46	0.150	74.14 ± 6.12	72.075 ± 6.58	1.78	0.078

**Table 2:** TLDA screening results of serum miRNA expression in macrosomia and controls.

MicroRNA	Case (*n* = 30)		Control (*n* = 30)		ΔΔCT
	CT	ΔCT	CT	ΔCT	
hsa-miR-1	36.01	14.06	33.97	11.97	2.09
hsa-miR-122	25.96	4.00	22.95	0.96	3.05
hsa-miR-192	27.97	6.02	25.97	3.97	2.04
hsa-miR-192-3p	35.98	14.03	33.99	11.99	2.04
hsa-miR-194	29.99	8.04	25.98	3.98	4.06
hsa-miR-296-5p	33.03	11.08	30.90	8.90	2.18
hsa-miR-301b	32.96	11.01	35.98	13.99	−2.98
hsa-miR-376a	31.98	10.03	29.98	7.99	2.04
hsa-miR-487b	34.00	12.05	32.00	10.00	2.04
hsa-miR-489	35.98	14.02	33.96	11.97	2.06
hsa-miR-500	36.01	14.05	33.99	12.00	2.05
hsa-miR-505	33.02	11.06	31.00	9.00	2.06
hsa-miR-518e	32.99	11.04	35.06	13.07	−2.03
hsa-miR-591	35.19	13.23	32.95	10.95	2.28
hsa-miR-625	35.97	14.01	33.96	11.97	2.05
hsa-miR-1262	35.00	13.04	32.96	10.97	2.07

**Table 3:** Individual qRT-PCR results (expression level) of serum miRNA expression in macrosomia and controls.

MicroRNA	Case (*n* = 30)		Control (*n* = 30)		*P* ^a^	*P* ^b^
	Mean	Median	Mean	Median		
has-miR-122	7.15 × 10^−2^	1.78 × 10^−2^	3.49 × 10^−2^	1.44 × 10^−2^	0.299	0.291
has-miR-192	3.35 × 10^−3^	2.51 × 10^−3^	3.56 × 10^−3^	3.04 × 10^−3^	0.792	0.691
has-miR-194	3.71 × 10^−4^	2.70 × 10^−4^	9.68 × 10^−4^	4.09 × 10^−4^	0.048^*^	0.045^*^
has-miR-296-5p	2.98 × 10^−4^	1.96 × 10^−4^	8.63 × 10^−4^	2.22 × 10^−4^	0.194	0.156
has-miR-376a	9.63 × 10^−5^	7.66 × 10^−5^	4.33 × 10^−4^	1.05 × 10^−4^	0.018^*^	0.016^*^
has-miR-487b	1.69 × 10^−4^	6.11 × 10^−5^	7.16 × 10^−4^	1.30 × 10^−4^	0.161	0.113
has-miR-505	5.03 × 10^−4^	2.71 × 10^−4^	1.84 × 10^−3^	4.33 × 10^−4^	0.232	0.176
has-miR-1262	1.40 × 10^−4^	8.40 × 10^−5^	6.95 × 10^−4^	1.01 × 10^−4^	0.112	0.095

^a^Univariate logistic regression models were used for analysis.

^b^Multiple logistic regression analyses adjusted for infant gender, maternal age, and maternal BMI when blood was collected.

∗Asterisks denote significant differences from controls (*P* < 0.05).

**Table 4:** Expression level of miR-376a and miR-194 in all participants.

MicroRNA	Case (*n* = 60)		Control (*n* = 60)		*P* ^a^	*P* ^b^
	Mean	Median	Mean	Median		
has-miR-376a	7.19 × 10^−5^	4.86 × 10^−5^	2.39 × 10^−4^	6.05 × 10^−5^	0.032^*^	0.034^*^
has-miR-194	5.37 × 10^−4^	2.54 × 10^−4^	6.70 × 10^−4^	3.29 × 10^−4^	0.497	0.521

^a^Univariate logistic regression models were used for analysis.

^b^Multiple logistic regression analyses adjusted for infant gender, maternal age, and maternal BMI when blood was collected.

∗Asterisks denote significant differences from controls (*P* < 0.05).
